# Emerging Non-Noble-Metal Atomic Layer Deposited Copper as Seeds for Electroless Copper Deposition

**DOI:** 10.3390/ma17071620

**Published:** 2024-04-02

**Authors:** Zihong Gao, Chengli Zhang, Qiang Wang, Guanglong Xu, Guoyou Gan, Hongliang Zhang

**Affiliations:** 1 Faculty of Materials Science and Engineering, Kunming University of Science and Technology, Kunming 650500, China; 2 Laboratory of Advanced Nano Materials and Devices, Ningbo Institute of Materials Technology and Engineering, Chinese Academy of Sciences, Ningbo 315201, China; 3 Ningbo Wakan Electronic Science Technology Co., Ltd., Ningbo 315475, China; 4 Center of Materials Science and Optoelectronics Engineering, University of Chinese Academy of Sciences, Beijing 100049, China

**Keywords:** Cu catalyst seeds, atomic layer deposition copper, electroless plating, copper printed circuits

## Abstract

Copper metal catalyst seeds have recently triggered much research interest for the development of low-cost and high-performance metallic catalysts with industrial applications. Herein, we present metallic Cu catalyst seeds deposited by an atomic layer deposition method on polymer substrates. The atomic layer deposited Cu (ALD-Cu) can ideally substitute noble metals Ag, Au, and Pd to catalyze Cu electroless deposition. The optimized deposition temperature and growth cycles of an ALD-Cu catalyzed seed layer have been obtained to achieve a flexible printed circuit (FPC) with a high performance electroless plating deposited Cu (ELD-Cu) film. The ELD-Cu films on the ALD-Cu catalyst seeds grown display a uniform and dense deposition with a low resistivity of 1.74 μΩ·cm, even in the through via and trench of substates. Furthermore, the ALD-Cu-catalyzed ELD-Cu circuits and LED devices fabricated on treated PI also demonstrate excellent conductive and mechanical features. The remarkable conductive and mechanical characteristics of the ALD-Cu seed catalyzed ELD-Cu process demonstrate its tremendous potential in high-density integrated FPC applications.

## 1. Introduction

Printed circuit boards (PCBs) serve as platforms containing electrical lines and pads that interconnect various electronic components to fulfill specific electrical or electronic functions [1]. The commercial production and application of PCBs commenced in the early 20th century and persists to play a pivotal role in electronics manufacturing today, representing a technology that is both traditional yet enduringly relevant. The further development of modern consumer electronics, smart wearable devices, and defense equipment has led to advancements in the integration and stability of cutting-edge PCB. A PCB integrates electronic components and sub-units, typically including lines, resistors, capacitors, diodes, and semiconductor chips. The conductive interconnect circuit on a PCB is a critical component for transmitting electrons, linking other elements chemically etched from the copper layer laminated between non-conductive substrates [2], such as flame-retardant 4 (FR-4) glass-reinforced epoxy laminate material. The basic circuitry design for flexible printed circuit (FPC) boards is similar to that of rigid PCBs. The primary distinction of FPC lies in the placement of flexible interconnected traces on flexible non-conductive substrates, such as polyimide (PI) or polyethylene terephthalate (PET). This surge in attention can be attributed to their exceptional and, in some respects, unparalleled characteristics, including remarkable flexibility, versatile shape adaptability, and lightweight construction [3].

In recent decades, numerous researchers have been making continuous endeavors to the surface and interface metallization of non-conductive materials such as FR-4 glass-reinforced epoxy laminate, PI, and PET substrates. It has been widely accepted in many aspects, and researchers have explored techniques including lamination, sputtering, screen printing, inkjet printing, electroplating, and electroless plating deposition (ELD) [4,5,6,7,8]. For example, antioxidant high-conductivity copper paste for the screen printing of low-cost flexible electronics under ambient conditions have been achieved using copper microflakes with surface passivation by formate ions and thiols [5]. Y. F. Wang et al. developed a compatible Ag^+^ complex and utilized a micro inkjet printing instrument to directly print on PET substrates. This process triggers the deposition of ultrafine copper patterns, each approximately 20 μm in width [6]. Although numerous innovative techniques or strategies for surface metallization have been reported, the practical application of these approaches in further miniaturized and integrated PCBs is hindered by limitations in the manufacturing processes. This is particularly the case for multi-layer high-density interconnection (HDI) PCB and FPC, which demand not only smaller line widths and spacing but also blind vias and through-holes with high aspect ratios between layers. Hence, the typical processes for commercializing PCBs still involve electroless deposition. Compared with the aforementioned emerging processes, the ELD process enables deposition within the through hole/blind via of HDI PCBs.

Cu electroless plating deposition stands as the preferred method for depositing interconnected conductors in cutting-edge nanoelectronics. The electroless copper layer can serve as a seed layer for electroplating, or it can be deposited onto the full metal thickness and minimizes redundancy. In an electroless plating process, metal ions in the plating bath are reduced to metal and deposited on the substrate to form a conductive pattern. The catalytic activity of the electroless copper deposition process is intricately related to the oxidation of the reducing agent present in the electroless bath [9]. This oxidation process serves as the rate-determining step, exerting direct influence on the overall efficiency of the deposition process. The electroless process commences with catalyst seeds on the insulating surface, which initiate the formation of metal nuclei [10]. The primary function of the surface catalyst is to enhance the dissociative adsorption of the reducing agent, leading to the formation of an adsorbed anion radical and adsorbed atomic hydrogen on the surface. The oxidation process of the adsorbed anion radical leads to the generation of an electron, while the adsorbed atomic hydrogen either undergoes recombination or becomes ionized. From there, the process becomes autocatalytic, with the metal serving as the site for the oxidation of the reducing agent. It seems that the typically precious metal components like Pd, Ag, and Au are commonly employed in the typical catalyst seeds for ELD [6,11,12]. The commercial catalyst established for electroless plating deposited Copper (ELD-Cu) production is the Sn/Pd nano-colloid, known for its dominant high catalytic activity in a wide variety of reducing agents [13,14]. Obviously, the high cost associated with these precious metal catalyst seeds significantly limits the potential applicability of the ELD process.

There has been increasing interest in using Cu catalyst seeds in the ELD process due to their cost-effectiveness and comparable catalytic activity. Several methods for catalyst seeds based on Cu metal have been developed towards the production of high-quality ELD-Cu films. Recently, the effectiveness of the emerging strategy for conductive copper patterns with low electric resistance on polyethylene terephthalate substrate by combining inkjet printing with copper nanoparticles conductive ink and electroless plating copper has been exemplified in a report by Y.B. Zhang et al. [15]. Additionally, Cu nanoparticles have been employed as catalyst seeds in conjunction with the reductant dimethylaminoborane (DMAB) in non-formaldehyde plating solution [16]. Despite these advancements, the introduction of Cu catalyst seeds in ELD-Cu still faces tough challenges, especially in achieving sufficient coverage of high aspect ratio microstructures (through via/trench).

In this context, atomic layer deposition (ALD) is experiencing increased interest in catalytic applications due to its precise thickness control at the Ångstrom level and extreme conformality on high aspect ratio structures [17]. For example, the atomic layer depositing ZnO thin films using Et_2_Zn (diethylzinc) and H_2_O as the precursors have been successfully used for photocatalysis [18]. In the field of atomic layer deposited Cu (ALD-Cu), the case reported here illustrates that continuous, smooth, and highly conformal Cu thin films have been achieved on a Si substrates below 120 °C by employing the strategy of utilizing the ligand-exchange reaction of Cu(dmap)_2_ (dmap dimethylamino-2-propoxide) with Et_2_Zn by a T-ALD process [19]. Recent cases reported by D.J. Hagen et al. also support the hypothesis that the introduction of ALD-Cu makes an island-like growth mode feasible in terms of conventional substrates, including SiO_2_, TaN, and carbon-doped oxide (COD) [20]. Interestingly there is not, to our knowledge, any study focused on ALD-Cu for ELD catalytic applications. The process of ALD-Cu has also been preliminarily conducted in our previous research endeavors [21,22], which offer an appetizing strategy for an ALD-Cu alternative to the conventional noble catalyst seeds. 

Herein, we propose a novel strategy that combines ALD-Cu and electroless plating techniques to create conductive copper patterns. An experimental demonstration of FPC based on both flexible PI and typical epoxy laminate substrates was carried out using ultra-thin ALD-Cu layers as the catalytic sites for the subsequent ELD-Cu. The findings for our results suggest that ALD-Cu layers enable a metallization of an epoxy laminate trench/via for applications of the copper patterns by means of the ALD-Cu-catalyzed ELD-Cu process. Furthermore, we have made an observation by applying ALD-Cu/ELD-Cu films and verified the excellent properties of the successively patterned flexible circuits and LEDs on treated PI substrates. The high adhesion strength of the ALD-Cu/ELD-Cu layer on PI substrates plays a critical role in determining high performance with noticeable resistance and remarkable fatigue resistance in these devices. The ALD-Cu catalyzed seed layer is a significant contributory factor to development of FPC.

## 2. Materials and Methods

**Materials.** Et_2_Zn and Cu(hfac)_2_ [copper (II)-hexafluoroacetylacetonate] with a purity of 99.9% were selected as the precursors for the ALD process and bought from AimouYuan (Nanjing, China). Commercially available polyimide slices (Kapton 100HN, DuPont, Wilmington, DE, USA) and copper clad epoxy laminates (FR4 Q100C, SYTECH, Dongguan, China) commonly utilized in FPCB and conventional PCB were used as the substrates. The difference between the substrates lies in their distinct surface chemistry. The laminate substrate is primarily composed of Si, Al, and Ca oxides, with composite formation facilitated by the epoxy, as shown in Figure S1. ALD processes exhibit self-limiting layer-by-layer growth on polar oxide surfaces, nearing an ideal state due to the presence of more reactive sites and heightened reactivity on the oxide surface (Figure S2). The PI substrates are known to be a non-polar surface with the lack of functional groups, large porosity, and free volume, yet it remains a significant challenge to develop ALD-Cu on polyimide substrates [22]. The typical commercial electroless plating solutions of Cu were purchased from JETEK Co., Ltd. (Wuxi, China). The trough holes and trenches were etched on epoxy laminate substrates by laser process and mechanical plating. Both the PI and epoxy laminate substrates were sequentially cleaned using deionized water and analytical grade (99.5%) absolute alcohol. 

An industry standard pre-treatment process (ISPP) for modifying the surface of the commercial FPC substrate was also applied to the PI slices for flexible circuits and device applications of ALD-Cu catalyzed ELD-Cu. The ISPP treatment involved the following steps: (1) A layer of copper foil was laminated onto the surface of the polyimide by a hot laminated method. (2) An etching solution (CuCl_2_, NaClO_3_, HCl) was used to remove the copper on the surface of the polyimide slices. (3) The PI slices were soaked in an aqueous solution of NaOH and blowing agents for neutralization and roughening. Deionized water and analytical grade (99.5%) absolute alcohol were used for sonication after each step.

**Preparation of Cu films.** The ALD-Cu catalyst seeds were deposited employing a commercial thermal-type ALD reactor equipment (TALD-150D, Kemicro, Jiaxing, China) with 6-inch reaction chamber. The Cu(hfac)_2_ and Et_2_Zn were heated to temperatures of 90 °C and 25 °C, respectively, and delivered to the reaction chamber with a N_2_ carrier gas. The N_2_ served as both the carrier and purging gas for the precursor vapor. The temperature of the pipeline was set to 110 °C to prevent the precursor from condensing in the pipeline. The substrates were placed in the center of the ALD reactor chamber and heated along with the chamber. The ALD process was initiated when the chamber temperature stabilized completely at the desired temperature. The temperatures of both the reaction chamber and the substrate remained stable during the reaction process. The ALD-Cu catalyst seeds were grown by a 500-cycle multi-pulse ALD-Cu process involving alternating exposures to Cu(hfac)_2_ and Et_2_Zn. The Cu(hfac)_2_ pulse/purge/Et_2_Zn pulse/purge time was 0.5 × 2/20/0.02/20 s. The detailed procedures of preparing ALD-Cu have been described in our previous work [21]. The base pressure of the ALD system was 0.0056 Torr, and during the deposition process with a nitrogen flow rate of 7 sccm, the pressure was maintained at 0.11 Torr.

For the electroless plating process of Cu, the ALD-seeded substrates were immersed immediately in a Cu ELD bath at room temperature for 30–120 min for the metallization. The electroless plating process of Cu was conducted at room temperature, without any additional heating or insulation measures. Afterward, the samples were rinsed in DI water and dried heat-treated at 60 °C for 30 min.

The electroless plated Cu films deposited on ISPP treated PI were patterned into Cu lines and flexible Cu circuits with the line widths ranging from 0.1 to 5 mm by a photolithography process. The as-made films Cu-PI was also designed with and applied to the flexible circuit. Two patch LEDs were welded to the circuit and passed through direct voltage of 3 V.

**Characterization and measurement.** X-ray diffraction (XRD, D8 Advance, Bruker, Billerica, MA, USA) was used to characterize the phase composition of Cu films. The Sheet resistance of various thin films was studied through four-point probe measurements (RTS-8, 4 Probes Tech, Guangzhou, China), and the average value was presented upon the test at least five times. The resistance of Cu lines was measured by a precision DC resistance meter (CHT3548, Zengjun, Shanghai, China). The morphology and grain size of the synthesized ALD-Cu islands or nanoparticles were determined by transmission electron microscopy (TEM, JEM2100, JEOL, Akishima, Japan) analysis. TEM samples were prepared by a direct growing of ALD-Cu onto carbon-coated molybdenum grids. Scanning electron microscopy (SEM, Verios G4 UC, Thermo Scientific, Waltham, MA, USA) with energy-dispersive X-ray spectroscopy (EDX) were used to observe the surface morphology and the thickness of the ELD-Cu films. The flexibility tester (HC-NS-9005, HC-test, Suzhou, China) was utilized to assess the bending fatigue resistance of the ALD-Cu-catalyzed ELD-Cu lines on treated PI.

## 3. Results and Discussions

**Figure 1:**
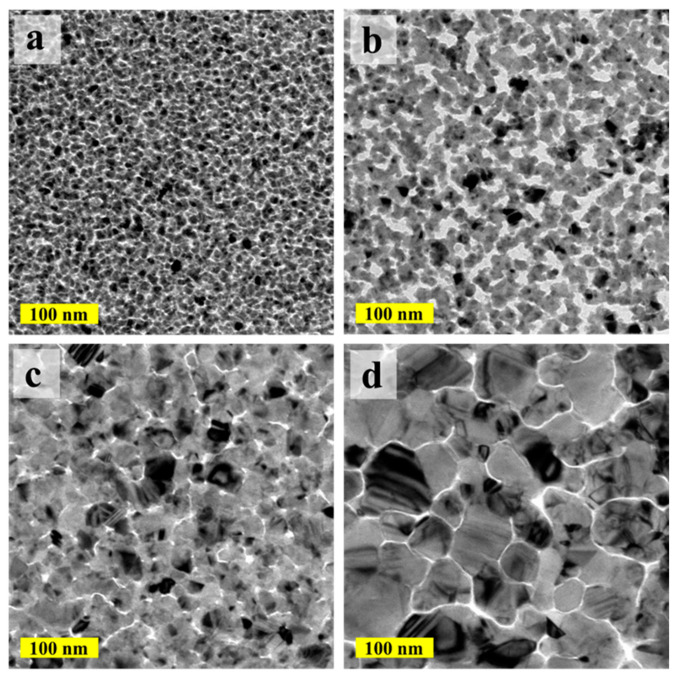
TEM images of the ALD-Cu islands/NPs deposited at (**a**) 110 °C, (**b**) 120 °C, (**c**) 130 °C, and (**d**) 150 °C for 250 growth cycles.

The morphology of the deposited ALD-Cu catalyst seeds under various depositing temperatures was examined with TEM, as shown in Figure 1. The ALD-Cu was found to have undergone island-type growth at the initial stage during the deposition (≤250 growth cycles in our case). The resulting thin film layer was discontinuous and exhibited non-conductive properties. In the case of the 110 °C deposited temperature (Figure 1a), numerous fine particles (≤10 nm) composed of homogeneous copper grains were displayed on the ALD-Cu seed layer. When the deposition temperature was increased to 120 °C, agglomerating Cu particles in the form of discontinuous and isolated islands were clearly observed. The copper islands were found to be partially connected, coalesced, and amalgamated at the deposited temperature of 130 °C, as depicted in Figure 1c. At a deposition temperature of 150 °C (Figure 1d), the ALD-Cu film demonstrated a notably larger discontinuous agglomeration, with island sizes exceeding 100 nm. The processing temperature range for ALD, or the so-called ‘ALD window’, was the region of nearly ideal ALD behavior between the nonideal regions. At higher temperatures, the surface species could decompose and allowed additional reactant adsorption. This behavior is similar to CVD by unimolecular decomposition [17]. The window temperature for ALD-Cu layer is known to range from 110 °C to 120 °C [21]. ALD-Cu is prone to partial CVD reaction at growth temperatures of 130 °C and 150 °C, which seems to be out of the temperature ‘window’, leading to an increased growth rate of ALD-Cu. Consequently, this results in higher copper loads at equivalent growth cycles. Moreover, the deposition of thin copper films is known to suffer from easy agglomeration, as the copper metal has rather poor adhesion to most substrates and the surface copper atoms are quite mobile at an elevated temperature [23]. The clear presentation in Figure 1 demonstrates the enhanced agglomeration effect of ALD-Cu particles as the deposition temperature rises, thereby leading to increased particle size of ALD-Cu. Based on the analysis above, it can be concluded that the increase in grain size and Cu load from 110 °C to 150 °C of ALD-Cu is attributed to the combined effects of CVD-like parasitic reactions and copper aggregation induced by the elevated deposition temperature [24].

**Figure 2:**
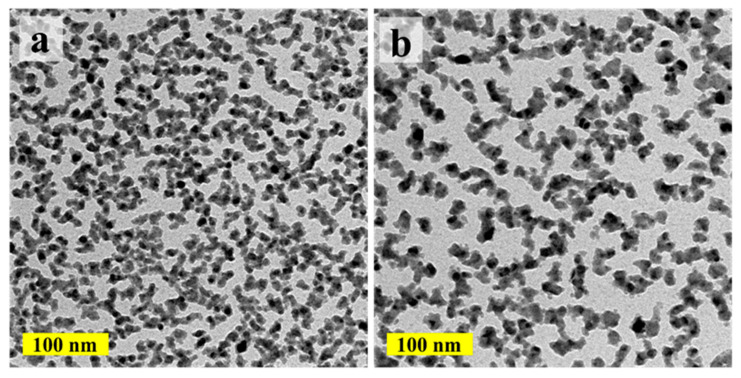
TEM images of the ALD-Cu islands/NPs deposited at 120 °C for (**a**) 150 and (**b**) 200 growth cycles.

The island size and Cu load of the ALD-Cu can also be regulated by adjusting the ALD growth cycles, thanks to the self-limited and layer-by-layer deposition characteristic of the ALD process [25]. Figure 2 shows the TEM images of the ALD-Cu islands/NPs deposited at 120 °C for 150 and 200 growth cycles compare with the 250-cycle growth sample in Figure 1b. As shown in Figure 2a, the ALD-Cu seeds deposited with 150 growth cycles are completely separated from each other and display a very fine grain size similar to the 250-growth cycle ALD-Cu seeds deposited at 110 °C. The ALD-Cu deposited with 200 cycles exhibits phenomena of mutual aggregation, accompanied by a reduction in the number of Cu islands, as shown in Figure 2b. As the growth cycles increase to 250 (Figure 1b), the island size of the ALD-Cu increases, and the number of copper islands also increases. The deposition of ALD-Cu involves the growth and coalescence of copper islands/NPs. ALD-Cu with varying growth cycles exhibits distinct island sizes and morphologies. According to Hagen et al. [20], the variation in island density (*N*) over time during growth can be elucidated by considering two concurrent processes, as delineated by the following Equation:

(1) $$\frac{dN}{dt}=U^{+}+U^{-}$$

*U*^+^ is the increase in island density that occurs due to the nucleation of islands from incoming atoms and *U*^−^ is the corresponding decrease caused by coalescence. It has been observed that the deposit efficiency and sheet resistance of the ELD-Cu film is influenced by the NPs size and NPs load of the catalyst seeds [16]. The freedom in the modulation of the island size and the load of Cu catalyst seeds on polymer substrates by adjusting the deposition temperature and growth cycles in the ALD-Cu process results in the optimal ALD-Cu catalyst seeds for the Cu electroless plating.

**Figure 3:**
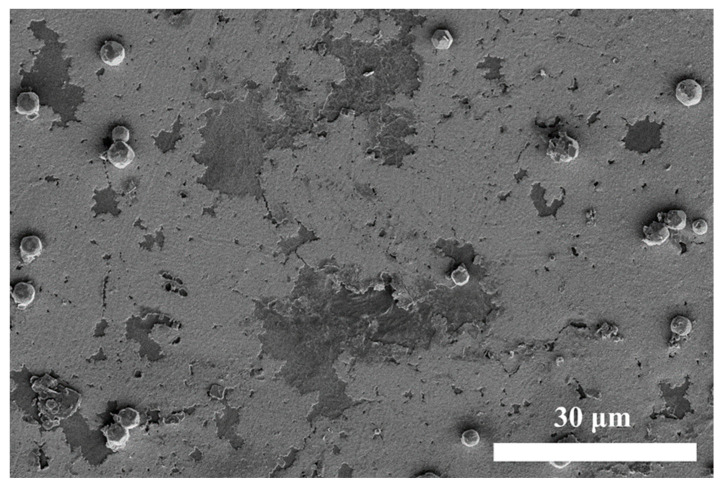
SEM image for the 60 min duration ELD-Cu catalyzed by the 120 °C ALD-Cu catalyst seed on PI substrates.

For electrocatalytic reactions, the particle size of metal catalysts can be tuned to modulate their catalytic properties [26]. In principle, smaller particle sizes generally exhibit better catalytic properties because of the larger surface area and high surface atoms that are shared. For example, the particle size of the Pd-Sn Colloid is generally in the range of 10 to 20 angstroms [27], which is the typical catalyst seed size in commercial ELD-Cu. In our case, the continuous ELD-Cu films catalyzed by the ALD-Cu catalyst seeds deposited at 130 °C and 150 °C were obtained (Figure S3a,b). In contrast, the ELD-Cu film catalyzed by ALD-Cu seeds deposited at 110 °C and 120 °C was observed to be discontinuous and partially detached, as shown in Figure S3c,d. Moreover, copper flakes and copper particles could be observed in the ELD-Cu plating solution of the ALD-Cu catalyzed seeds deposited at 110 °C and 120 °C. Further investigation was conducted on the sample catalyzed by ALD-Cu catalyst seeds deposited at 120 °C, as illustrated in the surface SEM image of Figure 3. The partial stripping of the electroless plated copper film was attributed to the weak adhesion between the lower temperature deposited ALD-Cu islands (exhibit smaller island size) and the substrate, primarily due to their inherently minimal interfacial contact area. In the production process of commercial flexible circuit boards, roughening the substrate is a fundamental method for enhancing the adhesion between the Cu film and the substrate film [28]. The roughened surface enhances the contact area between the copper film and the substrate, thereby forming a stronger adhesion. Based on these results, it can be inferred that the fine ALD-Cu catalyst seeds deposited at lower temperatures can dissolve or be disrupted during the vigorous electroless copper plating reaction, which happens before the formation of a continuous ELD-Cu film. Therefore, ALD-Cu deposited at temperatures of 130 °C and 150 °C are highly suitable as a catalyst seed for the ELD-Cu process.

**Figure 4:**
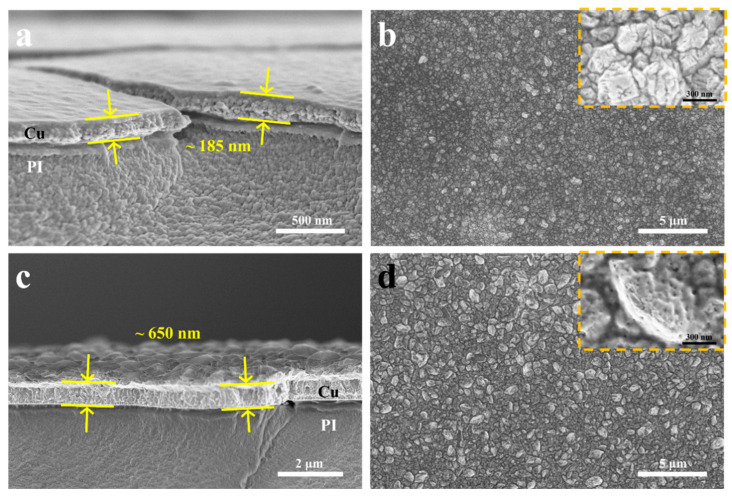
A comparison between SEM images of the surface and cross-sectional morphologies of the ELD-Cu films deposited on (**a**,**b**) 130 °C and (**c**,**d**) 150 °C ALD-Cu catalyst seeds. The insets are high-resolution enlarge surface SEM.

In the commercial printed circuit board fabrication process, the ELD process is initiated by the Pd/Sn catalyst on the insulating surface. Subsequently, the process transitions into an autocatalytic process where the copper film itself serves as the site for oxidation of the reducing agent. In other words, larger copper grains or a continuous copper film can also function as the catalyst seed layer for the ELD-Cu process. The formaldehyde reducing agent in commercial copper plating solutions is specifically chosen for the autocatalytic reaction of copper. Ohno et al. investigated the polarization behavior of several reductants on copper metal electrodes and concluded that copper exhibits the highest catalytic activity for formaldehyde oxidation compared to other common reductants [10]. SEM images in Figure 4 display the surface and cross-sectional morphologies of ELD-Cu films grown on ALD-Cu catalyst seeds with higher deposition temperatures (130 °C and 150 °C). It seems that the ELD-Cu films deposited on the 150 °C ALD seeds tend to become thicker (~650 vs. ~190 nm in Figure 4a,c) than the 130 °C ones. Interestingly, considering the surface morphologies of the ELD-Cu film, a very dense copper film with no obvious gaps between the crystals was obtained on the ALD-Cu seeds deposited at 130 °C, as depicted in Figure 4b. On the other hand, larger copper crystals with rough branch structured ELD-Cu were achieved when deposited on 150 °C ALD-Cu seeds (Figure 4d), along with a considerable multitude of pores. These pores are attributed to the generation of significant amounts of hydrogen gas under an excessively high deposition rate during the electroless deposition process [29]. The formaldehyde oxidation was accompanied by the evolution of H_2_ gas during the electroless deposition process on metals exhibiting positive free energy of hydrogen adsorption [30]:

(2) $${2H_{2}C\left(OH\right)O}^{-}+{2OH}^{-}\to {2HCO}_{2}^{-}+2H_{2}O+H_{2}↑+2e^{-}$$

The catalytic activity of Cu nanoparticles is usually associated with their specific surface area and loading capacity [16]. The thickness of the sample deposited at 150 °C was measured to be around 65 nm, almost twice that of the sample deposited at 130 °C, as shown in Figure S4. Furthermore, it was apparent that the discontinuous aggregated ALD-Cu seeds deposited at 150 °C possessed a larger specific surface area by combining the TEM images (Figure 1) of the ALD-Cu catalyst seed deposited at different temperatures. The higher deposition rate was exhibited in the ELD process for the 150 °C-deposited ALD-Cu catalyst seeds having larger specific surface areas and Cu loads, resulting in the formation of pores on the ELD-Cu surface. Therefore, although the deposition rate of the ELD-Cu increased as the deposition temperature of ALD catalyst seeds increased from 130 to 150 °C, the ELD-Cu film did not yield better conductivity because of the presence of pores between the branch structures on the copper surface. The resistivity of the ELD-Cu film on the 130 °C ALD seeds were measured to be approximately ~1.74 μΩ·cm, close to that of bulk Cu (1.7 μΩ·cm) [31]. In contrast, the resistivity of the ELD-Cu film grown on 150 °C-deposited ALD-Cu seeds was approximately 1.77 times greater than the bulk Cu. It can be concluded that the ELD-Cu film on 130 °C-deposited ALD-Cu catalyst seeds exhibited a relatively lower deposition efficiency but demonstrated improved resistivity and compactness.

**Figure 5:**
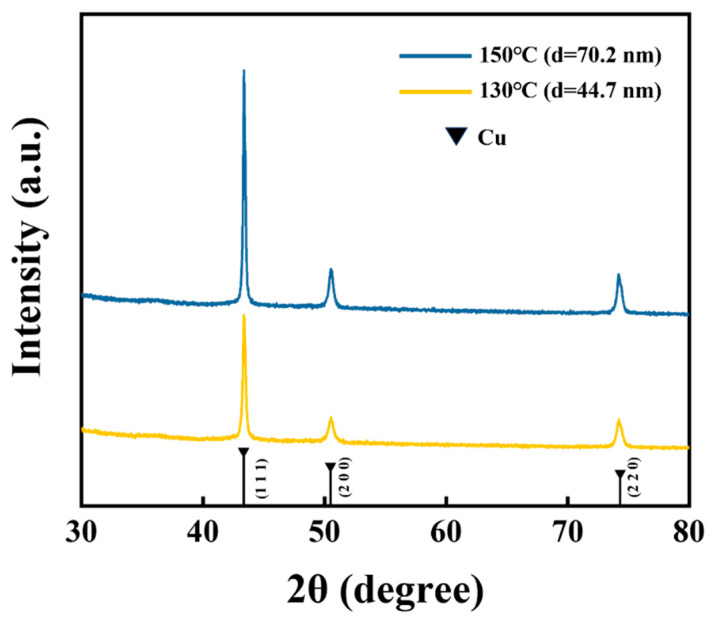
X-ray diffraction spectra of the ELD-Cu films deposited on ALD-Cu catalyst seeds. Powder Diffraction File (PDF) 04-0836 (Jade) is the standard spectrum of Cu metal.

Figure 5 displays the X-ray diffraction spectrum of the ELD-Cu films deposited on ALD-Cu catalyst seeds at different growth temperatures (130 and 150 °C). Only signals from Cu crystals were observed, with strong peaks corresponding to Cu(111) at 43.3° and two weak peaks for Cu(200) at 50.4° and Cu(220) at 74.1°. The peak intensity ratios for the Cu(111), Cu(200), and Cu(220) planes of the different ALD-Cu seeds catalyzed ELD-Cu films were shown to be 1:0.307:0.38 and 1:0.305:0.31, respectively. For those different samples, the variations in peak intensities were solely attributed to differences in film thickness, while their peak intensity ratios remained similar. Throughout the initial stages of the electroless plating process, the ELD-Cu film inherited the characteristics of the ALD-Cu catalyst seeds [7], with a dominant Cu(111) orientation. It seems to be inferred that the variations in the growth temperature of the ALD-Cu catalyst seeds had no influence on the growth orientation of ELD-Cu, but rather the deposition rate.

**Figure 6:**
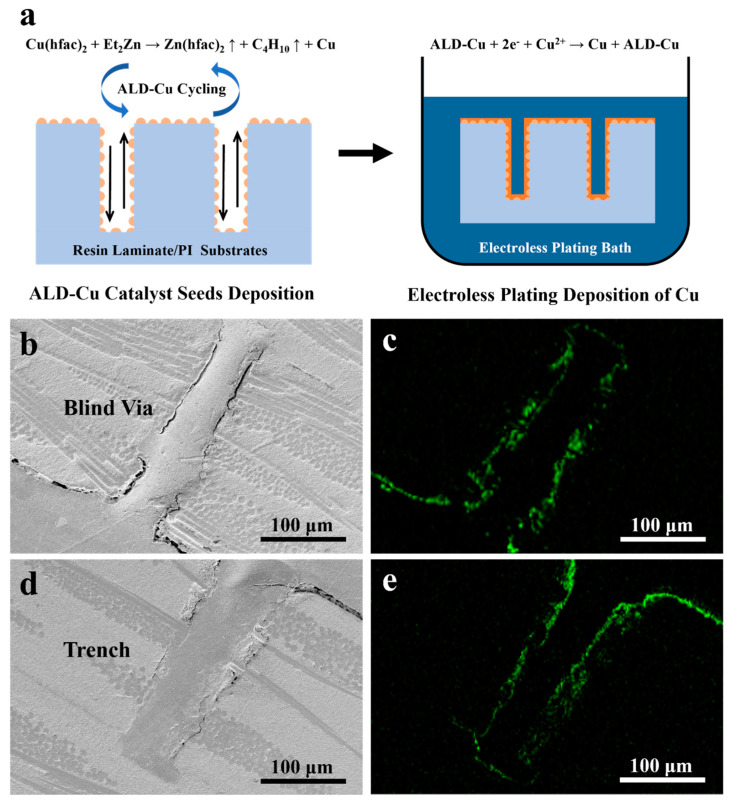
(**a**) General scheme for fabricating ALD-Cu catalyst seeds and ALD-Cu-catalyzed ELD-Cu. SEM images and EDS mapping for the 60 min duration ELD-Cu deposited on the (**b**,**c**) via and (**d**,**e**) trench of the epoxy laminate substrates.

In order to obtain conformal deposition during electroless plating, uniform distribution of the catalyst particles inside the trench/via is essential [32]. In our case, a 270 μm thick clad epoxy laminates with through vias (50 μm diameter) and trenches (80 μm width) were coated with ALD-Cu catalyst seeds, followed by Cu electroless plating. During the copper deposition process, no additional measures, such as mechanical vibration or bubble introduction, were utilized to enhance the ELD-Cu deposition. The general scheme for fabricating ALD-Cu-catalyzed ELD-Cu film on a via/trench is shown in Figure 6a. It should be noted that the internal spaces of the vias and trenches were filled with epoxy during the sample preparation process for SEM cross-section analysis. Figure 6b–e displays the SEM images and EDS mapping of the ELD-Cu deposited on the through via/trench of the epoxy laminate substrates for the 60 min duration. The EDS signals from the copper thin film can be obtained from both the bottom and sidewalls of the via/trench of the epoxy laminates substrates. The ELD-Cu film exhibited a conformal deposition inside the via and trench structures. The high step coverage of the ELD-Cu film was attributed to the introduction of conformal and continuous ALD-Cu process. The ALD process stood out for its capability to uniformly deposit conformal catalyst seeds within the vias/trenches of epoxy laminates. The self-limiting absorption and layer-by-layer deposition of ALD lead to the excellent step coverage and conformal deposition on high aspect ratio structures. There were only a finite number of surface sites, and the reactions could only deposit a finite number of surface species [17]. The deposition of ALD-Cu catalyst seeds occurred during alternating separate feeding of Cu and Zn precursors:

(3) $${Cu(hfac)}_{2}+{Et}_{2}Zn\to {Zn(hfac)}_{2}↑+C_{4}H_{10}↑+Cu$$

Once the continuous and conformal ALD-Cu catalyst seed was grown on the sidewalls and bottom of the via or trench, the subsequent Cu electroless plating process could be uniformly catalyzed and deposited. Overall, the introduction of ALD-Cu catalyst seeds proved to be an effective approach for copper interconnection in through vias, blind vias, and trenches, in combination with a conventional electroless plating.

**Figure 7:**
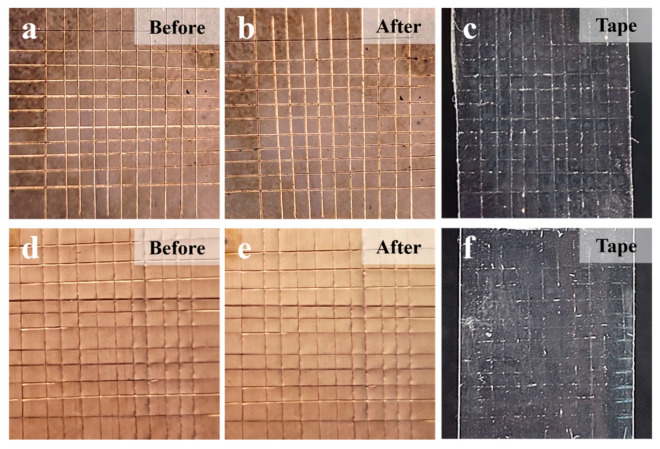
Optical images of ELD-Cu film deposited on different substrates before and after 3M tape test. (**a**–**c**) Cu film deposited on pristine PI and (**d**–**f**) Cu film deposited on treated PI.

A high adhesion strength between the ALD-Cu/ELD-Cu layer and PI substrate plays a critical role in ensuring the high performance of flexible electronics. A 3M tape test (Figure 7) was used to evaluate the adhesion strength between the Cu film and PI substrate [33]. The edges of ELD-Cu film were neatly cut and formed a square curve (side length = 1 mm) on the copper layer before 3M tape test, as shown in Figure 7a,e. Then, 3M 600-1PK tape was flatly pasted on the square curve of the copper layer and was torn off at a constant speed. After the 3M tape test, no Cu layer was observed to be removed from the pristine PI surface (Figure 7b) and none was left on the peeled tape (Figure 7c), except for cutting debris. These observations are consistent with the treated PI sample in Figure 7e,f. Based on ASTM D3359 standards [34], the bonding effectiveness of the ELD-Cu on PI substrates catalyzed by ALD-Cu was classified as level 5B, indicating a high degree of adhesion.

**Figure 8:**
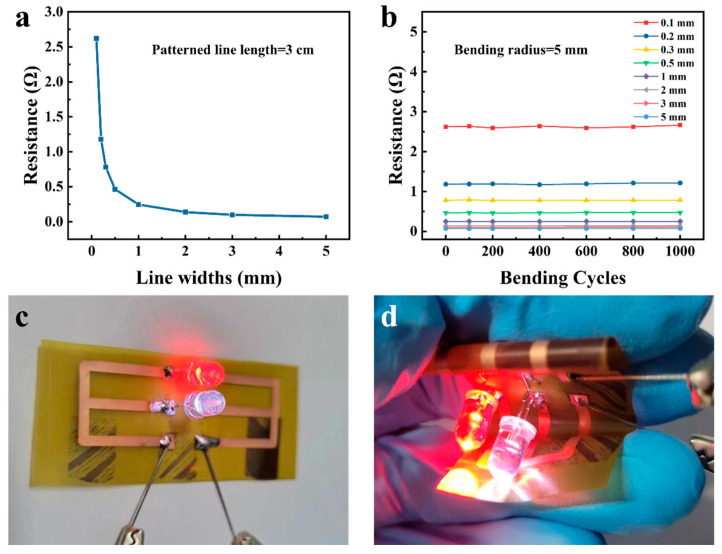
(**a**) Electrical resistance of the ALD-Cu-catalyzed electroless plating conductive lines as a function of line widths. (**b**) Relationship between Cu line resistance and cyclic bending at a given curvature radius of 5 mm. (**c**,**d**) Optical images showing a lighted LED device connected by patterned ALD-Cu-catalyzed ELD-Cu circuits.

The ALD-Cu/ELD-Cu films patterned based on industry standard pre-treatment process-treated PI substrates have exhibited uniform resistance distribution and significant resistance to bending. The resistance of the patterned Cu lines increased considerably as the width decreased, ranging from 0.072 Ω for a line width of 5 mm to 2.62 Ω for a line width of 0.1 mm, as displayed in Figure 8a. The resistance was found to decrease with the increased line width that fit well with the definition of electrical resistance, R = ρL/S, where ρ, L, and S represent the resistivity, length, and cross-sectional area, respectively, demonstrating the uniformity of the ALD-Cu-based EP-Cu conductors (Figure S5). The flexible performance of the patterned ELD-Cu lines was evaluated via bending tests with different widths (ranging from 0.1 to 5 mm). The relative electrical resistance of the patterned lines is plotted against bending cycles is shown in Figure 8b. These samples demonstrated excellent mechanical stability and durability after 1000 cycles of bending (180° cyclical bending tests), with a small curvature radius of 5 mm, even with the finest line width of 0.1 mm. The applicability and dependability of the ALD-Cu catalyzed ELD-Cu were confirmed through the fabrication of a flexible LED device on ISSP-treated PI substrates. The optical image in Figure 8c shows the LEDs lighted by a direct voltage of 3 V. Remarkably, these lighted LED can still function normally even when the ALD-Cu-catalyzed ELD-Cu lines are kneaded, as shown in Figure 6d. Therefore, by integrating ALD-Cu catalyst seeds with electroless plating process, the resulting conductive Cu films and lines enjoyed outstanding mechanical and conductive properties, making them suitable for the development of future Cu interconnections.

## 4. Conclusions

In summary, the ALD-Cu catalyst seeds were introduced into the Cu electroless plating process on epoxy and polyimide surfaces for high-density integrated Cu interconnection applications. By adjusting the deposition temperature and growth cycles of ALD-Cu, catalyst seeds for the electroless deposition of copper were obtained with controlled morphology and particle size. A 185 nm thick ELD-Cu film with a low resistivity of 1.74 μΩ·cm was achieved by immersing the ALD-Cu catalyst seed at 130 °C in a commercial electroless copper plating solution for 60 min. The XRD result demonstrates that the ELD-Cu films deposited on ALD-Cu catalyst seeds reveal a dominant Cu(111) orientation. This deposition remains consistent even in the high aspect ratio trenches and through the via of epoxy laminate substrates. Furthermore, the copper lines produced on flexible PI substrates demonstrated excellent bend resistance after 1000 bending test cycles at a small bending radius of 5 mm, signifying that the ALD-Cu catalyzed ELD-Cu patterns exhibit notable remarkable fatigue resistance. Flexible parallel-connected LED prototype devices were demonstrated on PI substrates using the ALD-Cu-catalyzed ELD-Cu process. The ALD-Cu catalyzed ELD-Cu process exhibited a novel approach to manufacturing high-quality conductive Cu without the use of noble metals, providing an alternative solution for commercial production of high-density copper interconnects and flexible electronics.

## Data Availability

Data are contained within the article and Supplementary Materials.

## Supplementary Materials

The following supporting information can be downloaded at: https://www.mdpi.com/article/10.3390/ma17071620/s1, Figure S1: SEM image (a) and elemental mapping results of (b) Si, (c) Al, (d) O, (e) C, and (f) Ca in the cross-sectional region of epoxy laminate substrates. Figure S2: (a) SEM cross image of the ALD-Cu seeded epoxy laminate substrates trench and (b) high resolution images. Figure S3: Optical image of the ELD-Cu films catalyzed by (a) 150 °C, (b) 130 °C, (c) 120 °C, and (d) 110 °C deposited ALD-Cu seeds on untreated PI surface. Figure S4: A comparison between cross-sectional SEM images of the ALD-Cu deposited at (a) 150 °C and (b) 130 °C. Figure S5: The resistance ratio and width between 0.1-mm-wide Cu line and others sample. References [21,35,36] are cited in the Supplementary Materials file.
