# Supplementary material for: Emerging Non-Noble-Metal Atomic Layer Deposited Copper as Seeds for Electroless Copper Deposition
Source: Materials (Basel). 2024 Apr 2;17(7):1620. doi: 10.3390/ma17071620 (PMC11012310; doi:10.3390/ma17071620)
Supplement: Supplementary file 1 [file materials-17-01620-s001.zip › materials-2883369-supplementary.pdf]

Supplementary Materials

Emerging Non-Noble-Metal Atomic Layer Deposited Copper as Seeds for Electroless Copper Deposition

Zihong Gao[1,2], Chengli Zhang[3], Qiang Wang[3], Guanglong Xu[3], Guoyou Gan[1,*], and Hongliang Zhang[2,4,*]

[1] Faculty of Materials Science and Engineering, Kunming University of Science and Technology, Kunming 650500, China

[2] Laboratory of Advanced Nano Materials and Devices, Ningbo Institute of Materials Technology and Engineering, Chinese Academy of Sciences, Ningbo 315201, China

[3] Ningbo Wakan Electronic Science Technology Co., Ltd., Ningbo 315475, China

[4] Center of Materials Science and Optoelectronics Engineering, University of Chinese Academy of Sciences, Beijing 100049, China

[*]Correspondence: ganguoyou@kust.edu.cn (G.G.); zhanghl@nimte.ac.cn (H.Z.);

Tel.: +86 574 86688153 (H.Z.) / Fax: +86 574 86688163 (H.Z.)

In this work, the ALD-Cu catalyst ELD-Cu is processed on polymer substrates, specifically focusing on polyimide and epoxy laminate substrates. These substrates are commonly utilized in FPCB and conventional PCB, respectively. The difference between the two substrates lies in their distinct surface chemistry, leading to varying difficulties in depositing the Cu catalyst seeds via ALD. Once the ALD-Cu catalyst seed is deposited, the ELD-Cu process can be carried out on both the PI and laminate substrates. Figure S1 illustrates the EDS elemental mapping of epoxy laminate substrates, showing that the substrate is primarily composed of Si, Al, and Ca oxides, with composite formation facilitated by the epoxy. Typically, ALD processes exhibit self-limiting layer-by-layer growth on polar oxide surfaces, nearing an ideal state due to the presence of more reactive sites and heightened reactivity on the oxide surface. As shown in Figure S2, ALD-Cu catalyst seeds can be deposited in a well-defined, continuous, and uniform manner at the bottom of the grooves of the composite particles on the epoxy laminate substrate. The PI substrates are known to be a non-polar surface with the lack of functional groups, large porosity, and free volume, yet it remains a significant challenge to develop ALD-Cu on polyimide substrates. [1] In other words, successfully implementing the ALD-Cu-based ELD-Cu process on a PI substrate suggests that this technique could be adapted for use with various other polymer materials. In our previous work, we have achieved high-quality low-temperature ALD-Cu thin films on PI substrates by improving the ALD-Cu precursor delivery process. [2] By leveraging an improved low-temperature ALD-Cu process, the ALD-Cu catalyst seeds can be deposited on both epoxy laminate substrates and PI, for the subsequent

electroless plating Cu deposition.

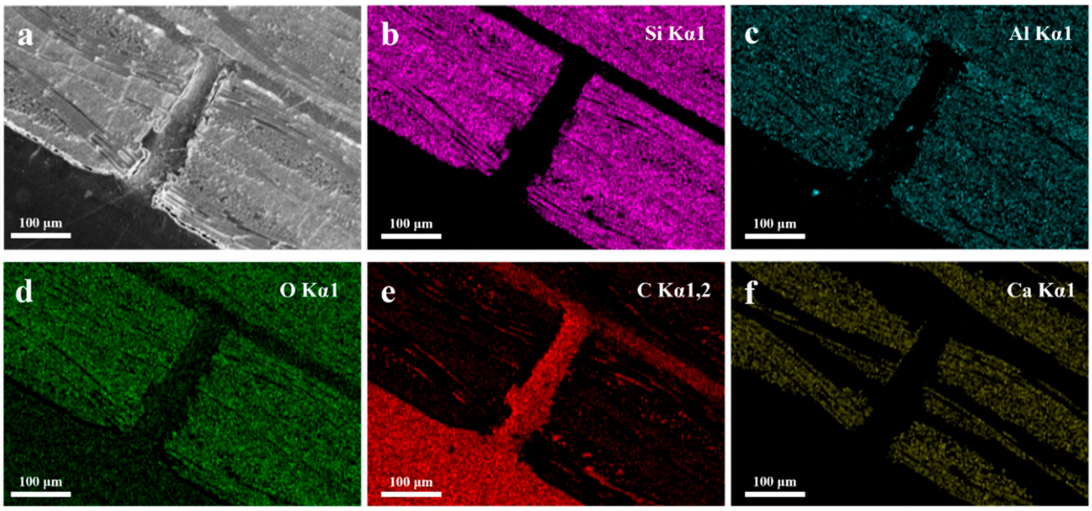

Figure S1: SEM image (a) and elemental mapping results of (b) Si, (c) Al, (d) O, (e) C, and (f) Ca in the cross-sectional region of epoxy laminate substrates.

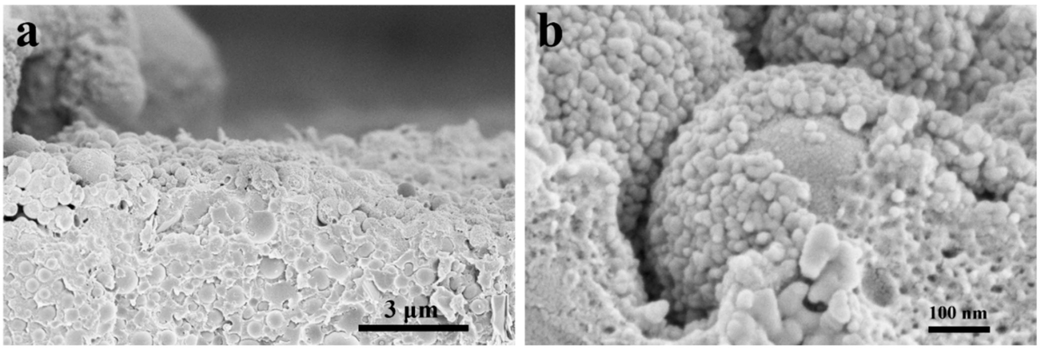

Figure S2: (a) SEM cross image of the ALD-Cu seeded epoxy laminate substrates trench and (b) high resolution images.

Figure S3 shows the optical image of the ELD-Cu films catalyzed by different ALD-Cu catalyst seeds on PI substrates. The continuous ELD-Cu films on ALD-Cu catalyst seeds deposited at 150°C and 130°C (Figure S3a and S3b) are obtained. In contrast, the ELD-Cu film catalyzed by ALD-Cu seeds deposited at 110 °C and 120 °C

is observed to be discontinuous and partially detached, as shown in Figure S3c and S3d. Copper flakes and copper particles can be observed in the ELD-Cu plating solution of the ALD-Cu catalyzed seeds deposited at 110 °C and 120 °C.

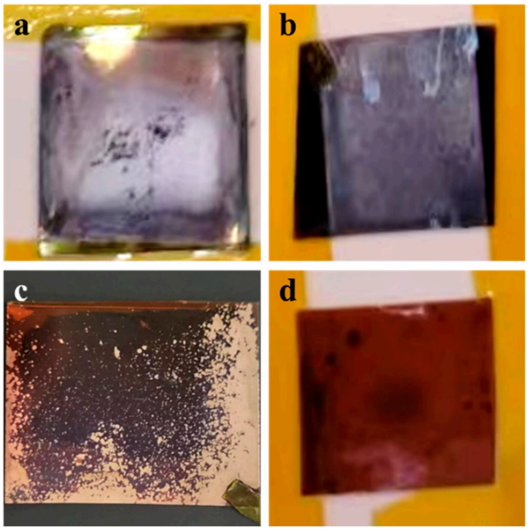

Figure S3: Optical image of the ELD-Cu films catalyzed by (a) 150 °C，(b) 130 °C, (c) 120 °C, and (d) 110 °C deposited ALD-Cu seeds on untreated PI surface.

Figure S4 presents cross-sectional SEM images of the ALD-Cu deposited at 150°C and 130°C. The thickness of the sample deposited at 150°C is measured to be around 65 nm, almost twice that of the sample deposited at 130°C.

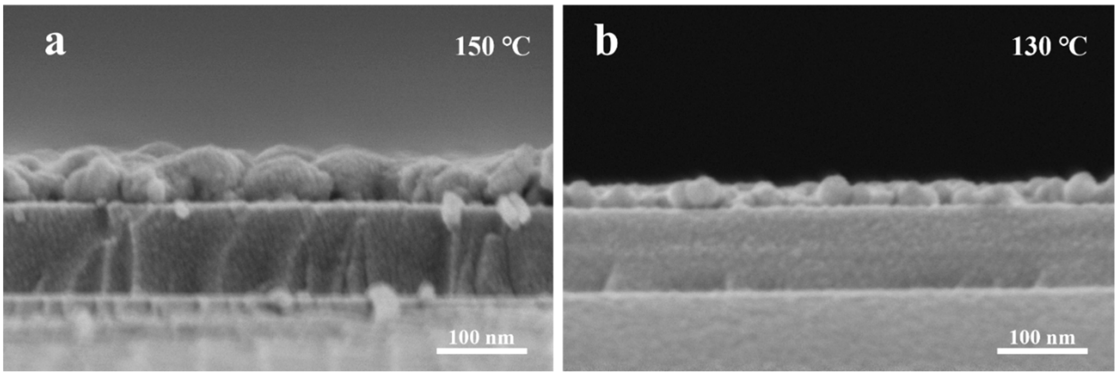

Figure S4: A comparison between cross-sectional SEM images of the ALD-Cu deposited at (a) 150 ℃ and (b) 130 ℃.

The resistance ratio and width between 0.1-mm-wide Cu line and others sample are exhibited in Figure S5. Obviously, the remarkable alignment of curves demonstrates a strong correlation between line width and resistance ratio, especially when the line width is less than 3 mm. For the 5-mm-wide Cu lines, the curve depicting the width ratio and resistance ratio deviates. These findings are consistent with the definition of electrical resistance, suggesting the uniformity of the ALD-Cu catalyzed ELD-Cu conductors. The deviation for the Cu line width over 3 mm can be ascribed to surface scattering and grain boundary scattering, arising from the larger width of the Cu line. [3]

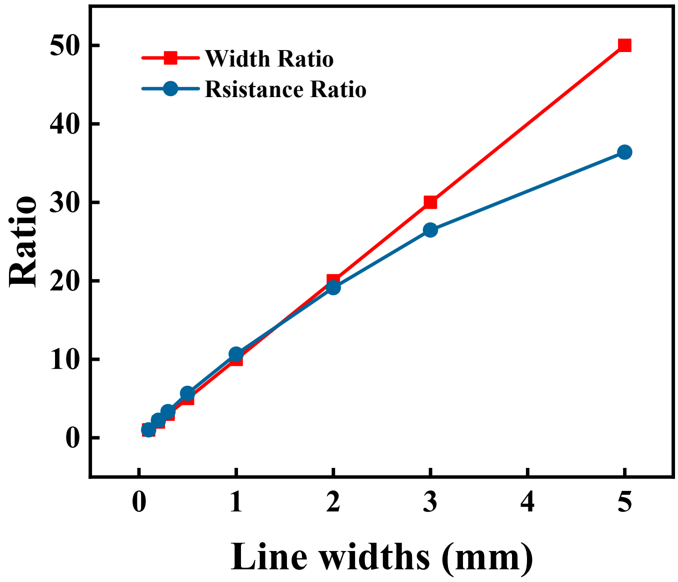

Figure S5: The resistance ratio and width between 0.1-mm-wide Cu line and others sample.

References

[1] G.N. Parsons, S.E. Atanasov, E.C. Dandley, C.K. Devine, B. Gong, J.S. Jur, K. Lee, C.J. Oldham, Q. Peng, J.C. Spagnola, P.S. Williams, Mechanisms and Reactions During Atomic Layer Deposition on Polymers, Coord. Chem. Rev. 257(23-24) (2013) 3323-3331.

[2] Z.H. Gao, C.L. Zhang, J.H. Gao, Q. Wang, G.L. Xu, H.T. Cao, Z.H. L., Nucleation and Growth of Low Resistivity Copper Thin Films on Polyimide Substrates by Low-Temperature Atomic Layer Deposition, Appl. Surf. Sci. 638(158072) (2023).

[3] T.H. Kim, X.G. Zhang, D.M. Nicholson, B.M. Evans, N.S. Kulkarni, B. Radhakrishnan, E.A. Kenik, A.P. Li, Large Discrete Resistance Jump at Grain Boundary in Copper Nanowire, Nano Lett. 10(8) (2010) 3096-3100.
